# Application of diffusion kurtosis imaging and ^18^F-FDG PET in evaluating the subtype, stage and proliferation status of non-small cell lung cancer

**DOI:** 10.3389/fonc.2022.989131

**Published:** 2022-09-30

**Authors:** Pengyang Feng, Zehua Shao, Bai Dong, Ting Fang, Zhun Huang, Ziqiang Li, Fangfang Fu, Yaping Wu, Wei Wei, Jianmin Yuan, Yang Yang, Zhe Wang, Meiyun Wang

**Affiliations:** ^1^ Department of Medical Imaging, Henan University People’s Hospital and Henan Provincial People’s Hospital, Zhengzhou, China; ^2^ Heart Center of Zhengzhou University People's Hospital, Henan Provincial People's Hospital, Zhengzhou, China; ^3^ Department of Orthopaedics, Henan University People’s Hospital, Zhengzhou, China; ^4^ Department of Medical Imaging, Zhengzhou University People’s Hospital and Henan Provincial People’s Hospital, Zhengzhou, China; ^5^ Department of Medical Imaging, Xinxiang Medical University Henan Provincial People’s Hospital, Zhengzhou, China; ^6^ Department of Medical Imaging, Henan Provincial People’s Hospital, Zhengzhou, China; ^7^ Central Research Institute, United Imaging Healthcare Group, Shanghai, China; ^8^ Beijing United Imaging Research Institute of Intelligent Imaging, United Imaging Healthcare Group, Beijing, China

**Keywords:** diffusion kurtosis imaging, diffusion-weighted imaging, ^18^F-FDG PET, non-small cell lung cancer, Ki-67

## Abstract

**Background:**

Lung cancer has become one of the deadliest tumors in the world. Non-small cell lung cancer (NSCLC) is the most common type of lung cancer, accounting for approximately 80%-85% of all lung cancer cases. This study aimed to investigate the value of diffusion kurtosis imaging (DKI), diffusion-weighted imaging (DWI) and 2-[^18^F]-fluoro-2-deoxy-D-glucose positron emission tomography (^18^F-FDG PET) in differentiating squamous cell carcinoma (SCC) and adenocarcinoma (AC) and to evaluate the correlation of each parameter with stage and proliferative status Ki-67.

**Methods:**

Seventy-seven patients with lung lesions were prospectively scanned by hybrid 3.0-T chest ^18^F-FDG PET/MR. Mean kurtosis (MK), mean diffusivity (MD), apparent diffusion coefficient (ADC), maximum standard uptake value (SUVmax), metabolic tumor volume (MTV) and total lesion glycolysis (TLG) were measured. The independent samples t test or Mann–Whitney U test was used to compare and analyze the differences in each parameter of SCC and AC. The diagnostic efficacy was evaluated by receiver operating characteristic (ROC) curve analysis and compared with the DeLong test. A logistic regression analysis was used for the evaluation of independent predictors. Bootstrapping (1000 samples) was performed to establish a control model, and calibration curves and ROC curves were used to validate its performance. Pearson’s correlation coefficient and Spearman’s correlation coefficient were calculated for correlation analysis.

**Results:**

The MK and ADC values of the AC group were significantly higher than those of the SCC group (all P< 0.05), and the SUVmax, MTV, and TLG values of the SCC group were significantly higher than those of the AC group (all P<0.05). There was no significant difference in the MD value between the two groups. Moreover, MK, SUVmax, TLG and MTV were independent predictors of the NSCLC subtype, and the combination of these parameters had an optimal diagnostic efficacy (AUC, 0.876; sensitivity, 86.27%; specificity, 80.77%), which was significantly better than that of MK (AUC = 0.758, z = 2.554, P = 0.011), ADC (AUC = 0.679, z = 2.322, P = 0.020), SUVmax (AUC = 0.740, z = 2.584, P = 0.010), MTV (AUC = 0.715, z = 2.530, P = 0.011) or TLG (AUC = 0.716, z = 2.799, P = 0.005). The ROC curve showed that the validation model had high accuracy in identifying AC and SCC (AUC, 0.844; 95% CI, 0.785-0.885);. The SUVmax value was weakly positively correlated with the Ki-67 index (r = 0.340, P< 0.05), the ADC and MD values were weakly negatively correlated with the Ki-67 index (r = -0.256, -0.282, P< 0.05), and the MTV and TLG values were weakly positively correlated with NSCLC stage (r = 0.342, 0.337, P< 0.05).

**Conclusion:**

DKI, DWI and ^18^F-FDG PET are all effective methods for assessing the NSCLC subtype, and some parameters are correlated with stage and proliferation status.

## Introduction

The morbidity and mortality rates of lung cancer have increased rapidly, and now it ranks first in incidence among malignant tumors (1); more than 80% of lung cancer cases are non-small cell lung cancer (NSCLC) (2). In recent years, the treatment of cancer has entered the era of precision medicine, and the subtype of NSCLC can affect the formulation of the treatment plan (3). Targeted therapy has led to significant improvements in adenocarcinoma (AC) patients, but for squamous cell carcinoma (SCC) patients, the treatment effect is poor, and serious complications may occur (4). For example, patients with SCC may be at risk of pulmonary hemorrhage when treated with bevacizumab therapy (5). In addition, clinical treatment is often determined according to the stage of lung cancer (6). Patients with early-stage NSCLC are mainly treated by surgery, followed by postoperative adjuvant radiotherapy and chemotherapy (7). Surgery is not recommended for patients with advanced-stage disease, and radiotherapy and chemotherapy are the main treatments (8, 9). As a nuclear antigen related to cell proliferation, Ki-67 can reflect the proportion of active cells in the cell cycle. Grant et al. (10) confirmed that the level of Ki-67 is closely related to proliferation, invasion, metastasis and prognosis of NSCLC (11). Needle biopsy is still the main method for obtaining histological characterization (12). In the past years, there has been a continuous development of novel technologies to improve diagnostic accuracy, reduce trauma and complications, for instance robot-assisted CT-guided lung biopsy has emerged as a safe technique in clinical practice (13), however, lung biopsies are still invasive and may result in pneumothorax, bleeding and/or other complications. 2-[^18^F]-Fluoro-2-deoxy-D-glucose positron emission tomography (^18^F-FDG PET) is a functional imaging technology that can reflect glucose metabolism in biological tissues (14, 15). Diffusion weighted imaging (DWI) is a noninvasive imaging technique widely used in clinical practice to evaluate the movement of water molecules, which can reflect the diffusion ability of water molecules in tissues (16). At present, ^18^F-FDG PET and DWI have been widely used in the identification of benign and malignant pulmonary nodules (17) and the evaluation of TNM stage of lung cancer (18). As an extension of DWI, diffusion kurtosis imaging (DKI) is a model based on the non-Gaussian distribution of water molecules, which is more sensitive to the diffusion of water molecules and more accurate in detecting tissue microstructure. Due to these advantages, DKI has been increasingly used for the evaluation of various diseases, such as prostate cancer (19), glioma (20), and cervical cancer (21). However, in the field of pulmonary lesion assessment, to our knowledge, only a few studies have explored the value of DKI in differentiating benign and malignant pulmonary nodules (22).

Therefore, this study aimed to investigate the value of DKI, DWI, and ^18^F-FDG PET in differentiating SCC and AC and to evaluate the correlation of each parameter with stage and proliferative status Ki-67, so as to provide new reference for relevant diagnosis and treatment of NSCLC.

## Materials and methods

### Study population

**Figure 1:**
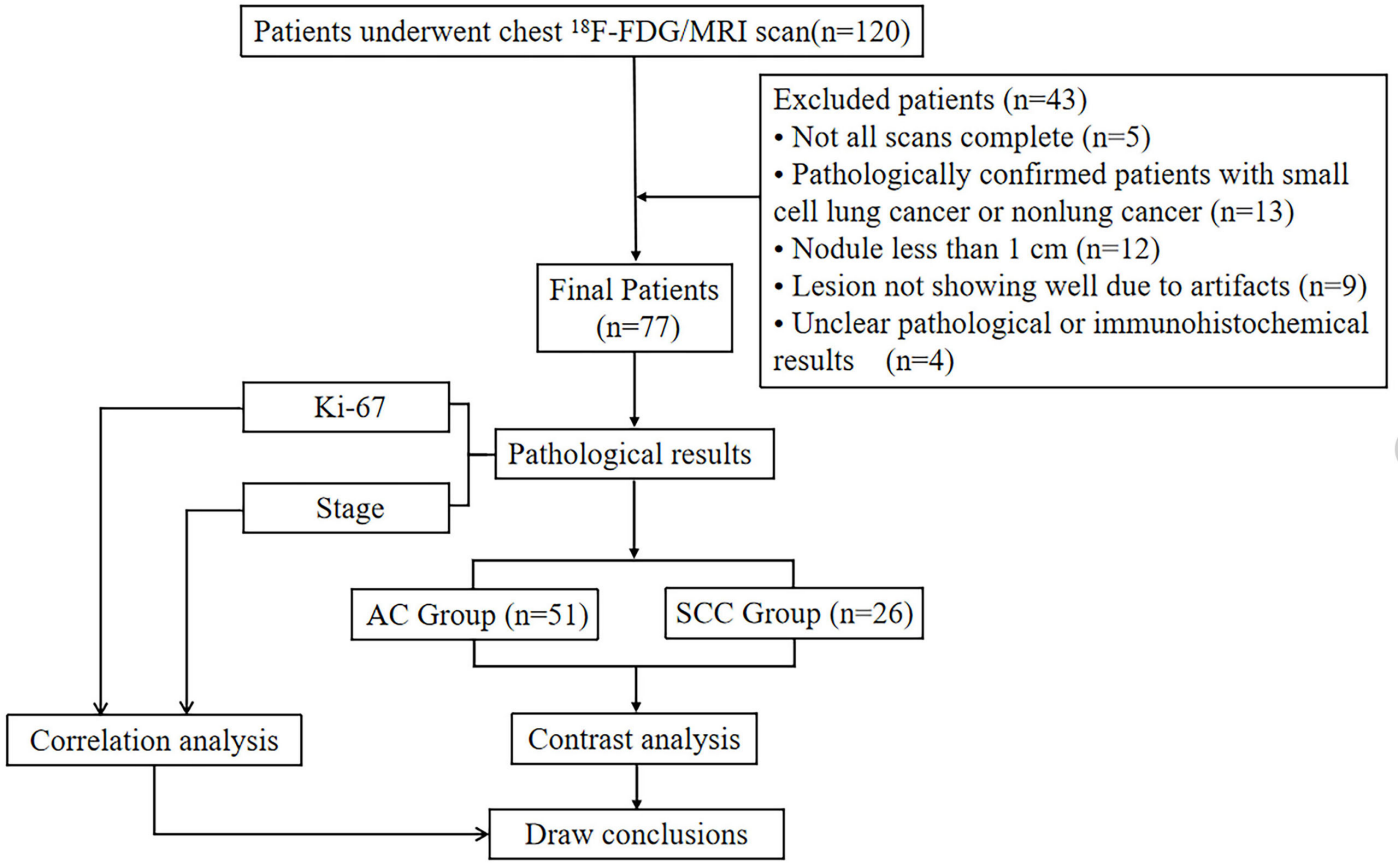
Flow diagram of the patient selection process.

This study was approved by the Research Ethics Committee (RCE) of our hospital. After receiving written informed consent from each patient, prospectively surveyed patients who were suspected of having a space-occupying lesion of the lung through CT examination in our hospital from July 6, 2020, to June 29, 2021, were enrolled. A total of 120 patients underwent chest ^18^F-FDG PET/MR scanning. The exclusion criteria were as follows: ① patients who failed to complete the scan due to claustrophobia or other physical discomfort (5 cases were excluded); ② patients with pathologically confirmed small cell lung cancer (4 cases excluded) or nonlung cancer (9 cases excluded) after ^18^F-FDG PET/MR; ③ patients with a nodule maximum diameter less than 1 cm (12 cases excluded); ④ patients with lesions not showing well due to artifacts (9 cases excluded) and ⑤ patients with unclear pathological or immunohistochemical results (4 cases were excluded). Ultimately, a total of 77 patients were included in this study. There were 48 males and 29 females, with an age of 62.73 ± 9.06 (range, 39 - 81) years, (**Figure 1**).

### Image acquisition

The study was conducted using a hybrid 3.0 T PET/MR scanner (uPMR790, UIH, Shanghai, China) with a 12-channel phased array body coil. The PET tracer ^18^F-FDG was produced by a cyclic accelerator and an automatic chemical synthesis system, and the radiochemical purity of the tracer was >95%. It was recommended that patients should not exercise vigorously within 24 hours before the examination and should fast for more than 6 hours to achieve a fasting blood glucose in the morning of< 7 mmol/L. The tracer was injected intravenously at the standard dose of 0.11 mCi per kilogram of body weight, and images were collected at a resting time of approximately 60 minutes (23) after injection. During the examination, the patient was placed in a supine position with his or her arms raised above the head. The scanning range was from the lung tip to the diaphragmatic surface of the lung bottom, and the whole lung was completely covered as the standard. MR-based attenuation correction (MRAC) was performed using a 3D T1-weighted spoiled gradient-echo sequence based on Dixon-based water-fat separation imaging (Wfi3d-trig). In MRAC processing, the corrected image is partitioned into four categories: soft tissue, fat, lung and air (24). PET scans were performed with one bed position and over an acquisition time of 27 minutes each. PET images were reconstructed using an ordered subsets expectation maximization (OSEM) algorithm (20 subsets, two iterations). In parallel with the PET scan, T1-weighted imaging (T1WI), T2-weighted imaging (T2WI), and DWI scans were performed first. Then, all the layers containing the lesions were selected from the DWI images, and their positions, layer thicknesses and layer spacing were copied into DKI for corresponding scanning. The detailed parameters of the MR sequence are shown in **Table 1**.

**Table 1:** MRI acquisition parameters.

Parameters	Wfi3d-trig	T1WI	T2WI	DWI	DKI
Sequence	2D - FSE	2D-FSE	2D-FSE	2D-SS-EPI	2D-SS-EPI
Orientation	Axial	Axial	Axial	Axial	Axial
TR/TE (ms)	4.92/2.24	3.54/1.51	3315/90.2	6000/65.6	2032/86
FOV (cm^2^)	50 × 35	40 × 30	38 × 30	40 × 30	50 × 35
Matrix	192 × 192	320 × 90	320 × 70	128×100	112×60
Slice thickness (mm)	2	5	5	5	5
Interval (mm)	0	1	1	1	1
NEX	2	1	2	1,4	1,4,8,8
Bandwidth (kHz)	/	650	260	2370	1630
b-values (s/mm^2^)	/	/	/	0,1000	0,500,1000,2000
Breath control	Breathe freely	Breath-holding	Breathing navigation	Breathe freely	Breathe freely
Scan time	2.04 min	14s	2.26min	1.30min	10.58min

Wfi3d-trig = 3D T1-weighted spoiled gradient-echo sequence with Dixon-based water-fat separation imaging; T1WI, T1-weighted imaging; T2WI, T2-weighted imaging; DWI, diffusion-weighted imaging; DKI, diffusion kurtosis imaging; FSE, fast spin-echo; SS-EPI, single shot echo-planar imaging; TR, repetition time, TE, echo time; FOV, field of view; NEX, number of excitation.

### Data post-processing

The collected PET/MR images were transferred to the United Imaging Workstation (uWS-MR: R005, UIH, Shanghai, China), and the workstation automatically performed image registration. For PET images, the PET/MR fusion software was used to post-process the metabolic parameters and automatically extract the volume of interest (VOI) of the tumor. Using 40% SUVmax as the threshold, the maximum standard uptake value (SUVmax), metabolic tumor volume (MTV) and total lesion glycolysis (TLG) of the tumor were automatically calculated. For DWI and DKI images, the parameters were processed using the workstation’s advanced analysis toolkit. The DWI parameter was generated using the following formulae:


(1) $$S_{b}/S_{0}=\;exp\;(-b\times ADC)$$

S_0_ is the signal strength when the b value is 0 s/m^2^, S_b_ is the signal strength at different b values, b is the diffusion sensitivity value, and ADC is the apparent diffusion coefficient. The DKI parameters were generated using the following formulae:


(2) $$S_{b}=\;S_{0}\times \;exp\;\left(-b\;\times \;D_{app}+\;b^{2}\times \;D_{app}2\times \;K_{app}/\;6\right)$$

Kapp represents the apparent kurtosis coefficient, Dapp represents the corrected apparent diffusion coefficient, and MD and MK are the mean Dapp and Kapp values of all directions. Using the PET/MRI fusion image as a reference, we manually delineated the ROIs (regions of interests) on the image of each layer containing the tumor on the axial T2WI image while paying attention to avoid places that are prone to artifacts, such as hemorrhage, necrosis and cystic degeneration. The software automatically copied the delineated ROIs to all parameter maps to calculate the average values based on the gross tumor volume (GTV). All the above quantitative parameters were independently processed and measured by two radiologists (PY.F and FF.F, 5 years and 14 years of experience) without any clinical or pathological information using a double-blind method.

### Histopathologic analysis

All patients in the study provided pathological specimens, which were obtained by focal biopsy or surgical procedures within 4.19 ± 2.06, (range 1-7) days after the PET/MR scan. The tumor tissue was isolated and sent to the pathology department of our hospital to determine the NSCLC subtype. Formalin fixation, dehydration, wax immersion, embedding, sectioning and routine HE staining were performed. The eighth edition of International Association for the Study of Lung Cancer (IASLC) was used for staging classification (25). The expression of Ki-67 was analyzed by immunohistochemistry with a mouse anti-human Ki-67 monoclonal antibody (MIB-1, DAKO, Denmark). Brown−yellow granules or brown granules in the nucleus were regarded as positive cells, 5 high-power fields were selected, and 500 cells were randomly detected in each section for observation.

### Statistical analysis

SPSS 23.0 software and MedCalc 15.0 software were used for data analysis. The intraclass correlation coefficient (ICC) was used to assess the consistency of each parameter by two radiologists, as follows: r ≥ 0.75, excellent agreement; 0.60 ≤ r< 0.75, good agreement; 0.40 ≤ r< 0.60, fair agreement; and r< 0.40, poor agreement (26). The Shapiro–Wilk test was used to test whether the data in each group conformed to a normal distribution. The Mann–Whitney U test or independent samples t test was used to compare the differences in each parameter between the AC and SCC groups. Parameters conforming to normal distribution were expressed as mean ± standard deviation, and parameters conforming to non-normal distribution were expressed as median (interquartile range). The Bonferroni method was used for correction analysis. Receiver operating characteristic (ROC) curves were used to assess the diagnostic efficacy of each parameter value (alone or in combination). The Delong test was used to determine whether the area under the ROC curve (AUC) of each parameter (alone or in combination) was different. Logistic regression analysis was used for the evaluation of independent predictors. A control model was built by bootstrapping (1000 samples); the model was tested with multiple regressions, and its performed was verified with calibration curves, decision curve analysis (DCA), and ROC curves. The correlation between each parameter and Ki-67 index was analyzed according to the Pearson correlation coefficient. Spearman’s correlation coefficient was used to analyze the correlation between each parameter and NSCLC stage. The test standard was set to 0.05, and the difference was considered statistically significant at P< 0.05.

## Results

### Features of all patients

The clinical and pathological characteristics of all the patients are shown in **Table 2**.

**Table 2:** Clinicopathologic features of the patients.

Characteristics	Data
Age (years), mean ± SD	62.73 ±9.06
Gender, N (%)	
Male	48 (62.34)
Female	29 (37.66)
Maximum diameter (mm), mean ± SD	3.36±1.51
Smoker , N (%)	
Yes	39 (50.65)
No	38 (49.35)
CEA ng/ml, mean ± SD	11.31±20.02
CA-199 KU/l, mean ± SD	31.95±61.29
CA-125u/ml, mean ± SD	38.12±72.73
NSCLC subtype, N (%	
AC	51 (66.23)
SCC	26 (33.77)
IASLC stage (2015), N (%)	
IA1 IA2 IA3 IB	0 (0.00) 4 (5.19) 4 (5.19) 1 (1.30)
IIA IIB IIIA	2 (2.60) 8 (10.39) 4 (5.19)
IIIB IIIC	17 (22.08) 7 (9.09)
IVA IVB	14 (18.18) 16 (20.78)

AC, adenocarcinoma; SCC, squamous cell carcinoma; CEA, carcinoembryonic antigen; CA-199, carbohydrateantigen-199; CA-125, carbohydrateantigen-125; NSCLC, non-small cell lung cancer; IASLC, International Association for the Study of Lung Cancer.

### Consistency test

The MK, MD, ADC, SUVmax, MTV and TLG values measured by the two observers all had high consistency, and the ICCs were 0.826, 0.799, 0.819, 0.823, 0.869 and 0.757, respectively. The average of the two observers’ parameter values was taken as the final result and included in this study.

### Differences in parameters

**Figure 2:**
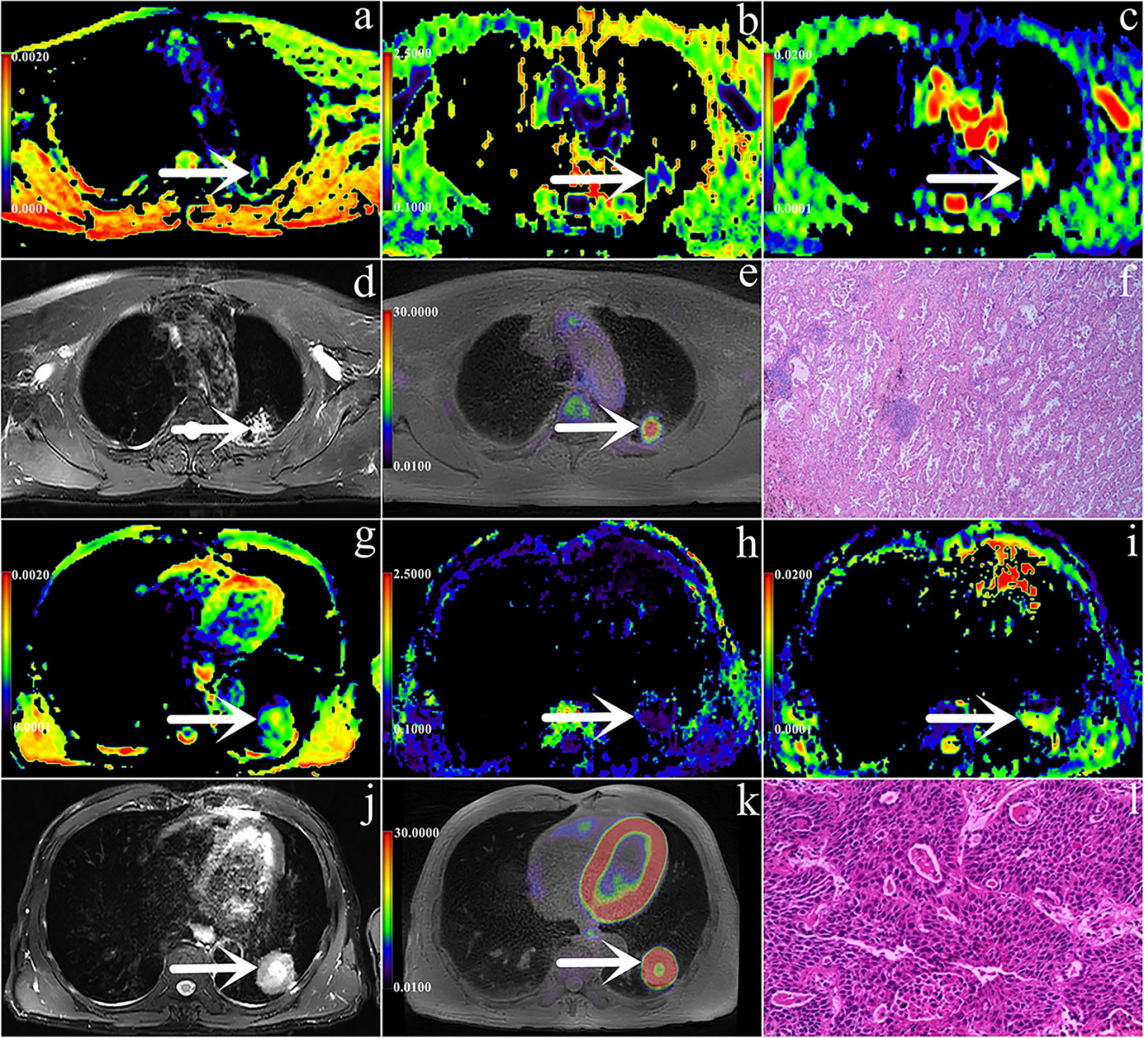
**(A–L)** are PET/MR images and pathological images. **(A–F)** Male, 56 years, with staging IIB, AC (shown by white arrows), Ki-67 is 30%, ADC = 1.505 × 10^-3^mm^2^/s, MK = 0.652, MD = 2.768 × 10^-3^mm^2^/s, SUVmax = 5.08 g/cm^3^, MTV = 2.44 cm^3^, TLG = 7.28 g; g-l Male, 58 years, with staging IIIB, SCC(shown by white arrows), Ki-67 is 70%, ADC = 1.068 × 10^-3^mm^2^/s, MK = 0.291, MD = 2.622 × 10^-3^mm^2^/s, SUVmax =11.84 g/cm^3^, MTV = 12.248 cm^3^, TLG = 83.408 g. In these images, a/g are ADC pseudo-colored maps, b/h are MK pseudo-colored maps, c/i are MD pseudo-colored maps, d/j are T2WI images, e/k are PET images and f/l are pathological images (magnification=100).

**Figure 2** shows the pseudocolor diagram of DKI, DWI and ^18^F-FDG PET parameters. The MK and ADC values in the AC group were significantly higher than those in the SCC group [0.55 (0.47, 0.73) (range, 0.22 - 2.14) vs. 0.42 (0.32, 0.53) (range, 0.23 - 0.71), 95% CI (0.080 - 0.228); (1.39 ± 0.16) (range, 1.05 - 1.77)×10^-3^ mm^2^/s vs. (1.26 ± 0.21) (range, 0.83 - 1.66)×10^-3^ mm^2^/s, 95% CI (0.032 - 0.294); (P< 0.001, P = 0.008)], while the values of SUVmax, MTV and TLG in the SCC group were significantly higher than those in the AC group [12.00 (8.39, 15.00) (range, 4.87 - 28.77) g/cm^3^ vs. 7.25 (5.03, 11.26) (range, 0.84 - 19.48)g/cm^3^, 95% CI (1.830 - 6.440); 14.84 (7.65, 35.83) (range, 2.39 - 96.64) cm^3^ vs. 6.50 (1.99, 14.65) (range, 0.40 - 86.98) cm^3^, 95% CI (2.878 - 13.000); 83.97 (40.85, 140.08) (range, 1.90 - 661.98) g vs. 27.17 (5.32, 77.00) (range, 0.37 - 447.96) g, 95% CI (19.770 - 74.248); (all P = 0.001, 0.002 and 0.002, respectively)]. There was no significant difference in the MD value between the AC group and SCC group [3.45 (2.95, 4.28) (range, 1.31 - 10.62) ×10^-3^ mm^2^/s vs. 3.26 (2.01, 3.84) (range, 1.12 - 8.68) ×10^-3^ mm^2^/s, 95% CI (0.116 - 1.060); (P = 0.122)], (**Table 3**).

**Table 3:** Comparison of different parameters in subtypes of NSCLC.

Parameters	AC	SCC	t/z value	Mean /Median difference	95% confidence interval	P value	Corrected P value
MK	0.55 (0.47,0.73)	0.42 (0.32,0.53)	-3.689	0.153^a^	0.080 - 0.228	< 0.001^c^	0.0001^d^
MD(×10^-3^mm^2^/s)	3.45 (2.95,4.28)	3.26 (2.01,3.84)	-1.546	0.461^a^	0.116 - 1.060	0.122^c^	0.732
ADC (×10^-3^mm^2^/s)	1.39 ± 0.16	1.26 ± 0.21	0.275	0.118	0.032 - 0.294	0.008^b^	0.048^d^
SUVmax (g/cm^3^)	7.25 (5.03,11.26)	12.00 (8.39,15.00)	-3.431	4.035^a^	1.830 - 6.440	0.001^c^	0.006^d^
MTV (cm^3^)	6.50 (1.99,14.65)	14.84 (7.65,35.83)	-3.075	7.034^a^	2.878 - 13.000	0.002^c^	0.012^d^
TLG (g)	27.17 (5.32,77.00)	83.97 (40.85,140.08)	-3.086	38.618^a^	19.770 - 74.248	0.002^c^	0.012^d^

NSCLC (non-small cell lung cancer), AC (adenocarcinoma), SCC (squamous cell carcinoma), MK (mean kurtosis), MD (mean diffusivity),ADC (apparent diffusion coefficient),SUVmax (maximum standard uptake value), MTV (metabolic tumor volume), TLG (total lesion glycolysis).

a Independent sample Hodges-Lehmann to get the median difference.

b Normally distributed data; Comparisons were performed by independent t-test; Results expressed as mean ± SD.

c Non-normally distributed data; Comparisons were performed by Mann–Whitney U test; Results expressed as median and interquartile range (in parentheses).

95% confidence interval for mean difference and median difference.

d Corrected P value < 0.05 was considered to have passed the Bonferroni test correction.

### Regression analyses

Age, sex, smoking, maximum diameter, location, lobulation sign, spicule sign, pleural depression sign, stage, CEA, CA-199, C-125 and related parameters were included in logistic regression analysis. Univariate analysis showed that sex, smoking, maximum diameter, stage, MK, ADC, SUVmax, TLG, and MTV were predictors of NSCLC subtype (all P< 0.1). Multivariate analysis showed that MK, SUVmax, TLG and MTV were independent predictors of NSCLC subtype (P = 0.005, 0.014, 0.025 and 0.015, respectively) (**Table 4**).

**Table 4:** Univariate and multivariate analyses were performed on factors associated with NSCLC.

Parameters	Univariate Analyses		Multivariable Analyses	
	OR (95% CI)	P value	OR (95% CI)	P value
AC vs SCC				
Age (year)	1.004 (0.952 - 1.058)	0.892	/	/
Sex	3.733 (1.219 - 11.438)	0.021	5.391 (0.630 - 46.108)	0.124
Smoker	4.207 (1.498 - 11.819)	0.006	4.095 (0.817 - 20.510)	0.086
Maximum diameter (mm)	1.605 (1.113 - 2.316)	0.011	1.198 (0.577 - 2.489)	0.628
Location	1.664 (0.636 - 4.355)	0.300	/	/
Lobulation sign	1.965 (0.754 - 5.119)	0.167	/	/
Spicule sign	0.759 (0.294 - 1.957)	0.568	/	/
Pleural depression sign	0.462 (0.170 - 1.253)	0.129	/	/
Stage	1.278(1.031 - 1.583)	0.025	1.301 (0.913 - 1.853)	0.146
CEA ng/ml	0.974 (0.939 - 1.010)	0.152	/	/
CA-199 ku/l	1.005 (0.997 - 1.013)	0.209	/	/
CA-125u/ml	1.001 (0.995 - 1.008)	0.651	/	/
MK	0.001(0.000 - 0.070)	0.001	0.000 (0.000 - 0.021)	0.005
MD(×10^-3^mm^2^/s)	0.775 (0.535 - 1.124)	0.179	/	/
ADC(×10^-3^mm^2^/s)	0.024 (0.001 - 0.430)	0.011	0.013 (0.000 - 1.282)	0.064
SUVmax(g/cm^3^)	1.208 (1.075 - 1.357)	0.002	1.484 (1.084 - 2.031)	0.014
MTV(cm^3^)	1.026 (0.999 - 1.054)	0.060	1.120 (1.015 - 1.235)	0.025
TLG(g)	1.004 (1.000 - 1.008)	0.052	0.976 (0.958 - 0.995)	0.015

All factors with P < 0.1 in the univariate analyses were included in the multivariate regression analyses. The influencing factors of P < 0.05 were considered as independent predictors. OR, odds ratio; CI, confidence interval. OR (odds ratio), CI (confidence interval), NSCLC (non-small cell lung cancer), AC (adenocarcinoma), SCC (squamous cell carcinoma), MK (mean kurtosis), MD (mean diffusivity), ADC (apparent diffusion coefficient), SUVmax (maximum standard uptake value), MTV (metabolic tumor volume), TLG (total lesion glycolysis).

### Comparison of diagnostic efficacy

The AUCs of MK, SUVmax, TLG, MTV and ADC used to diagnose SCC and AC were 0.758, 0.740, 0.716, 0.715 and 0.679, respectively (all P< 0.05), but there was no statistically significant difference in the diagnostic efficacy among these parameters (all P > 0.05). The cutoff values for each parameter were as follows: MK: 0.43, ADC: 1.270 ×10^-^3 mm2/s, SUVmax: 7.27 g/cm^3^, TLG: 19.11 g and MTV: 5.05 cm^3^.

**Figure 3:**
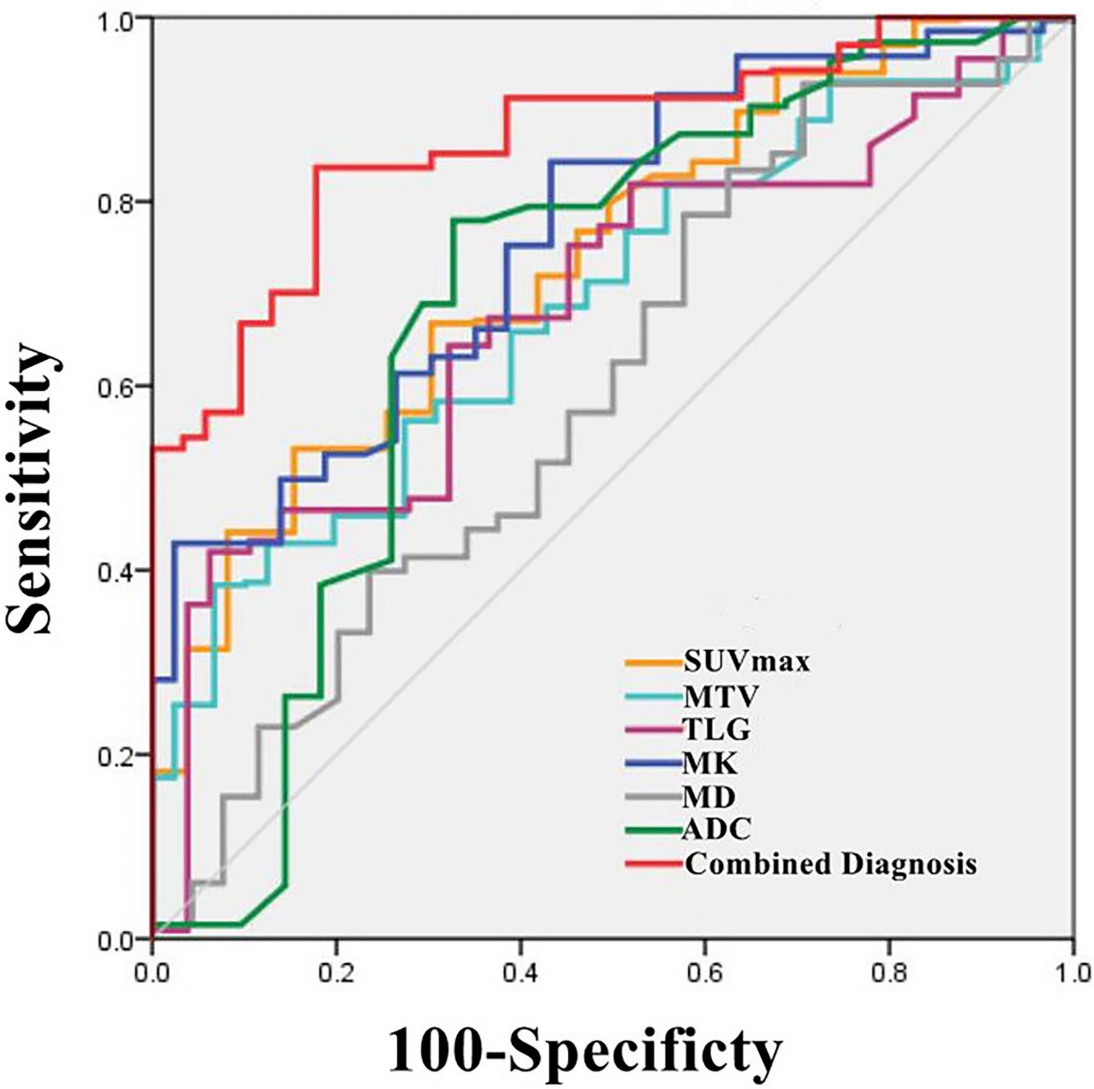
Graph shows ROC curves of different parameters (SUVmax, MTV, TLG, MK, MD and ADC) and the combination of independent predictors (MK+SUVmax+MTV+TLG) for discriminating AC group and SCC group (AUC was 0.740, 0.715, 0.716, 0.758, 0.608, 0.679 and 0.876 respectively).

Independent predictors MK, SUVmax, TLG and MTV were used for combined diagnosis, and the AUC (MK + SUVmax + TLG + MTV) was 0.876 (sensitivity, 86.27%; specificity, 80.77%; P< 0.001). Moreover, the difference between AUC (MK + SUVmax + TLG + MTV) and AUC (MK), AUC (ADC), AUC (SUVmax), AUC (MTV), AUC (TLG) was significant (Z = 2.554, 3.322, 2.584, 2.530, 2.799; all P< 0.05) (**Figure 3**, **Table 5**).

**Table 5:** ROC Analysis of the diagnostic performance for different parameters and methods alone or in combination for differentiating Adenocarcinoma (AC) from Squamous Cell Carcinoma (SCC).

Parameters	AUC (95%CI)	P value	Cutoff	Sensitivity	Specificity	Youden
MK	0.758(0.647-0.849)	< 0.001	0.43	84.31%	57.69%	42.01%
MD(×10^-3^mm^2^/s)	0.608(0.490-0.718)	0.1230	/	/	/	/
ADC(×10^-3^mm^2^/s)	0.679(0.563-0.781)	0.0139	1.270	76.47%	65.00%	41.86%
SUVmax (g/cm^3^)	0.740(0.628-0.834)	< 0.001	7.27	52.94%	84.62%	37.56%
MTV(cm^3^)	0.715(0.601-0.812)	< 0.001	5.05	45.10%	92.31%	37.41%
TLG(g)	0.716(0.602-0.813)	< 0.001	19.11	49.02%	92.31%	41.33%
Combined Diagnosis	0.876(0.781-0.940)	< 0.001	/	86.27%	80.77%	67.04%

AUC (area under the curve), CI (confidence interval), MK (mean kurtosis), MD (mean diffusivity), ADC (apparent diffusion coefficient), SUVmax (maximum standard uptake value), MTV (metabolic tumor volume), TLG (total lesion glycolysis), Combined Diagnosis means the combination of independent predictors (MK, SUVmax, MTV and TLG).

### Model validation

**Figure 4:**
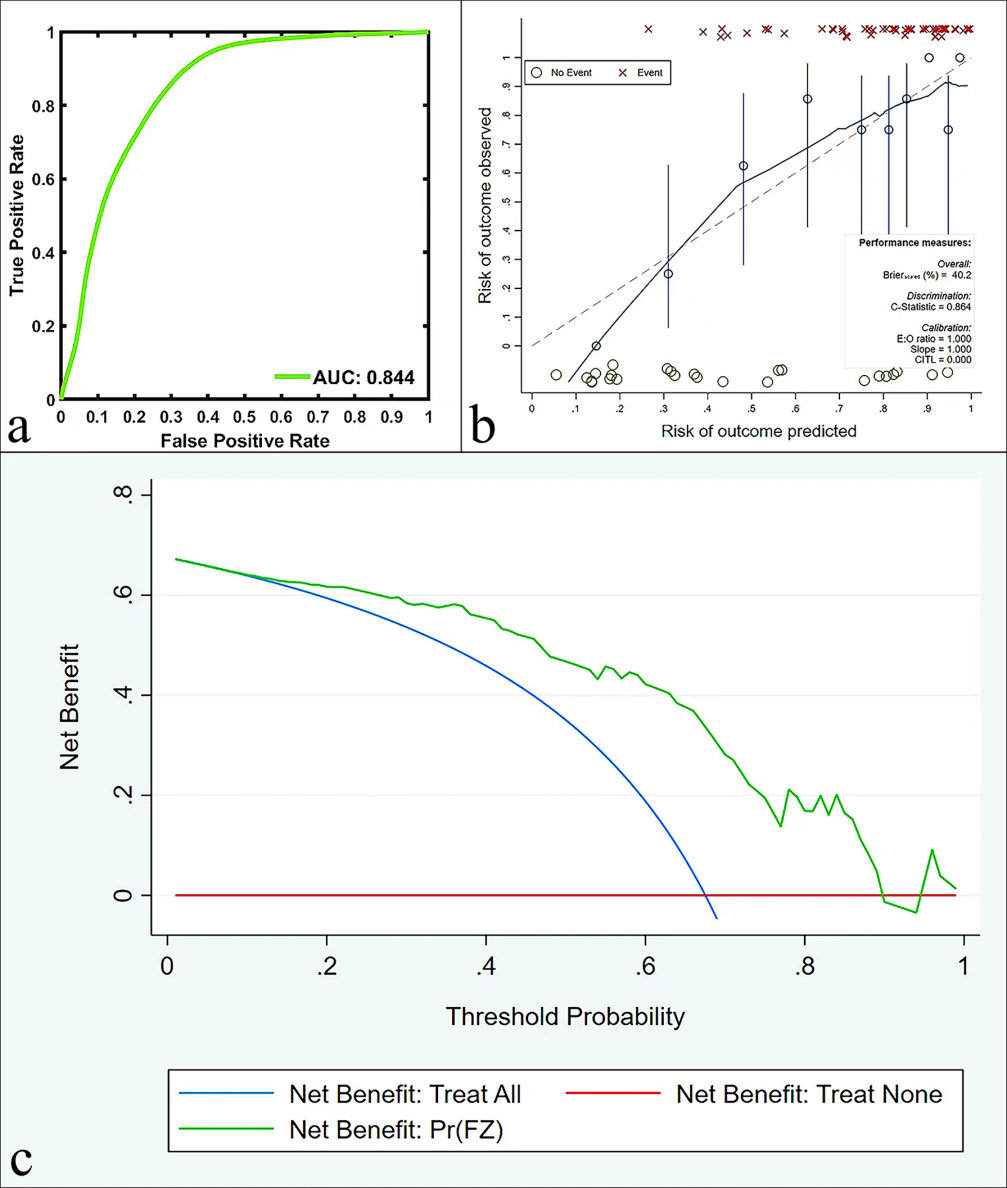
ROC curves **(A)**, calibration curves **(B)**, and DCA **(C)** in the validation model for predicting AC and SCC.

Bootstrapping with 1000 samples was used to validate the multivariate regression model. The ROC showed that the validation model had high accuracy in identifying AC and SCC (AUC, 0.844; 95% CI, 0.785-0.885; **Figure 4A**). The calibration curves indicated that the validation model was highly consistent with the original model (C-statistic, 0.864, **Figure 4B**). DCA showed that the model could provide a high net benefit for relevant patients (**Figure 4C**).

### Correlation analysis

There was a weak positive correlation between the SUVmax value and the Ki-67 index (r1 = 0.340, P< 0.05). ADC and MD values were weakly negatively correlated with the Ki-67 index (r2 = -0.256, r3 = -0.282, all P< 0.05). There was no significant correlation between MTV, TLG, MK and the Ki-67 index (all P > 0.05). The differences between r1 and r2, r2 and r3, and r1 and r3 were not significant (Z = 0.561, 0.171, 0.391, all P > 0.05) (**Table 6**).

**Table 6:** Correlation of each parameter with Ki-67 and stage.

Parameters	Ki-67			NSCLC stage		
	r	P value	Corrected P value	r	P value	Corrected P value
MK	−2.223	0.052	0.312	−0.089	0.442	2.652
MD(×10^-3^mm^2^/s)	−0.282	0.013	0.078	−0.016	0.888	5.328
ADC (×10^-3^mm^2^/s)	−0.256	0.025	0.150	−0.139	0.227	1.362
SUVmax (g/cm^3^)	0.340	0.002	0.012^a^	0.187	0.104	0.624
MTV (cm^3^)	0.148	0.198	1.188	0.342	0.002	0.012^a^
TLG (g)	0.116	0.315	1.890	0.337	0.003	0.018^a^

CAC (adenocarcinoma), SCC (squamous cell carcinoma), MK (mean kurtosis), MD (mean diffusivity), ADC (apparent diffusion coefficient), SUVmax (maximum standard uptake value), MTV (metabolic tumor volume), TLG (total lesion glycolysis).

a Corrected P value < 0.05 was considered to have passed the Bonferroni test correction.aptions.

There was a weak positive correlation between the MTV and TLG values and NSCLC stage (r4 = 0.342, r5 = 0.337, all P< 0.05), and there was no significant difference between r4 and r5 (Z = 0.034, P > 0.05). There was no significant correlation between the SUVmax, ADC, MK, and MD values and NSCLC stage (all P > 0.05) (**Table 6**).

## Discussion

### Diffusion-related parameters to assess the NSCLC subtype, stage, and proliferative status

ADC and MD all reflect the restricted diffusion of water molecules in the tissue and are closely related to the cell density, intracellular matrix and number of organelles (27). In this study, both ADC and MD values were weakly negatively correlated with the Ki-67 index, and these results are similar to those of Peng et al. (28), further indicating that ADC and MD can be used for the assessment of Ki-67. One explanation is that the higher the Ki-67 index is, the stronger the tumor invasive ability, the higher the cell density, and the higher the nucleocytoplasmic ratio (29), which leads to the restricted diffusion of intracellular and extracellular water molecules, which in turn leads to a decrease in ADC and MD values (30). This study further explored the value of ADC and MD in evaluating NSCLC stage, and the results showed that there was no significant correlation between ADC, MD and NSCLC stage, which was similar to the results of Paul’s study (31). The reason may be that NSCLC stage is comprehensively judged by many factors, such as the size of the primary focus, the depth of infiltration, the adjacent tissue involvement range, and the presence or absence of lymph node metastasis. ADC and MD mainly reflect cell density, so it seems unlikely that tumor stage could be accurately predicted only according to ADC and MD values. In terms of identification between SCC and AC, previous studies have shown that the cells are more densely packed in SCC than in AC (32), so the diffusion of water molecules in SCC is more restricted and the ADC is reduced, which is consistent with the results of this study. However, in terms of MD, SCC and AC showed no significant difference. We speculate that this may be because MD reflects the average value of ADC in all directions, so the variation in different lesions is not as significant as ADC in a single direction. In addition, the choice of b values and number of different b values applied in DKI may also affect the diagnostic performance of MD (33).

MK is the most representative parameter of DKI (34), and its value is closely related to the complexity of the tumor tissue structure. The more complex the structure is, the more significant the deviation of the water molecule diffusion motion from the Gaussian distribution, and the larger the MK value (35). In a report on cervical cancer, Meng et al. (21) proposed that the MK value of SCC was significantly higher than that of AC, perhaps because cervical SCC has a compact structure and less mucus secretion capacity than AC. However, our results showed that lung AC had a higher MK value than lung SCC. This may be related to the high number of AC cases in this study, and advanced-stage AC accounted for the majority of AC cases. Advanced tumor tissue structure is more complex and heterogeneous (21), thus leading to higher MK in AC. In addition, more air-containing tissues in the lungs may also be one of the reasons for the differences in the above research results. In the assessment of Ki-67, our study showed no correlation between the MK value and the Ki-67 index. However, a study by Peng et al. (28) for advanced-stage lung adenocarcinoma suggested that with the increase in Ki-67 expression, the tumor cell atypia and the complexity of the tissue structure increased, and the MK value increased. The reason for the above differences may be that this study includes both SCC and AC, and the b value parameters of DKI are different from those used by Peng et al. (28). In the future, we will optimize the research and scanning parameters and further explore the value of MK and Ki-67 in NSCLC. In addition, this study also found no correlation between MK and stage, suggesting that MK may not be used for the evaluation of NSCLC.

### Metabolic-related parameters to assess the NSCLC subtype, stage, and proliferative status

SUVmax is the most commonly used semiquantitative diagnostic index in clinical practice and reflects the level of glucose metabolism in the most active part of the tissue (36). For tumor lesions, previous studies have shown that the higher the malignant degree of tumor cells is, the faster the proliferation rate, and the higher the uptake of ^18^F-FDG is, the greater the SUVmax value (37). In this study, SUVmax was significantly different between the SCC group and the AC group (30), which is basically consistent with the research results of Port et al. (38). Compared with AC, SCC cells have a shorter doubling time, a faster proliferation rate (39, 40), and increased ^18^FDG uptake, which leads to increased SUVmax. Higher GLUT-1 protein expression in SCC (41) and differences in blood perfusion (42, 43) may also contribute to the increased SUVmax. MTV mainly reflects the metabolic volume of the tumor, and TLG mainly reflects the metabolic activity of the overall tumor (44). This study revealed that there were significant differences in MTV and TLG values between the SCC group and AC group, which was similar to the results of Koh et al. (40). On the one hand, this result may be related to the differences in cell proliferation, GLUT-1 expression and blood perfusion between SCC and AC (42, 43); on the other hand, the larger lesion volume of patients with SCC in this study may have contributed to the change in MTV and TLG values. In this study, NSCLC stage showed a weak positive correlation with MTV and TLG but no correlation with SUVmax, indicating that MTV and TLG values can better predict NSCLC stage. Although SUVmax is the most commonly used parameter, it only represents the biological characteristics of a single dimension of the tumor and is highly sensitive to noise (44). In fact, for advanced-stage tumors, the composition, shape, and uptake of ^18^F-FDG are uneven. Recent studies have also confirmed that MTV and TLG reflect the biological information of tumors more comprehensively, especially in the assessment of lung cancer stage (45). We also found that the SUVmax value was weakly positively correlated with the Ki-67 index, indicating that SUVmax can initially predict proliferation status. The reason may be that a high Ki-67 index often means that tumor cells have strong proliferation ability and vigorous metabolism (29), so their glucose uptake increases and SUVmax value increases. This study further analyzed the correlation between MTV, TLG and Ki-67, but the results showed that there was no significant correlation between them and Ki-67 expression. This is inconsistent with previous studies (46, 47). We speculate that this may be because there is still a difference between the tumor volume calculated by ^18^F-FDG PET/MR and the actual volume in this experiment, so it is difficult to accurately reflect the tumor metabolic burden.

### Diagnostic efficacy comparison

In terms of comparison of diagnostic efficacy, we found that the AUC of the combined model of independent predictors MK, SUVmax, MTV and TLG was higher than that of any single parameter. We speculate that this may be because the combined model integrates the advantages of each parameter, so it can better evaluate the characteristics of lesions. Therefore, if conditions permit, the comprehensive use of multiple imaging methods to evaluate patients may yield the greatest benefit.

### Limitations

There are several limitations to this study. First, sample size of this study was limited, and AC and advanced-stage samples accounted for a large proportion. Second, patients with tumors less than 1 cm in diameter and respiratory motion artifacts that made the image unclear were excluded. Third, there are currently few studies on DKI sequences in the lungs. No standards have yet been established for choosing a suitable b-value and ensuring a suitable signal-to-noise ratio at a high b-value, so it is still challenging to determine the best values for lesion evaluation.

## Conclusion

DKI, DWI, and metabolism-related parameters can be used to evaluate the NSCLC subtype, stage, and Ki-67 index. The combination of MK, SUV, MTV and TLG may be a potential imaging marker to differentiate SCC from AC.

## Data availability statement

The raw data supporting the conclusions of this article will be made available by the authors, without undue reservation.

## Ethics statement

The studies involving human participants were reviewed and approved by ethics committee of Henan Provincial People’s Hospital, Approval No. 2020116. The patients/participants provided their written informed consent to participate in this study.

## Author contributions

Study concepts and design, MW. Literature research, PF, TF,and ZS. Clinical studies, ZH, ZL and BD. Data analysis, FF, YW, ZS and ZW. Manuscript preparation, PF, WW and BD. Manuscript editing, JY, YY and ZS. All authors contributed to the article and approved the submitted version.

## Funding

This work was supported by the National Key R&D Program of China (2017YFE0103600), the National Natural Science Foundation of China (81720108021 and 31470047), the Zhengzhou Collaborative Innovation Major Project (20XTZX05015), Zhongyuan Thousand Talents Plan Project - Basic Research Leader Talent (ZYQR201810117), the Key Project of Henan Province Medical Science and Technology Project (LHGJ20210001 and LHGJ20210005) and Henan provincial science and technology research projects (212102310689).

## Acknowledgments

We acknowledge financial support by the National Natural Science Foundation of China.

## Conflict of interest

Authors JY, YY, and ZW were employed by United Imaging Healthcare (UIH).

The remaining authors declare that the research was conducted in the absence of any commercial or financial relationships that could be construed as a potential conflict of interest.

## Publisher’s note

All claims expressed in this article are solely those of the authors and do not necessarily represent those of their affiliated organizations, or those of the publisher, the editors and the reviewers. Any product that may be evaluated in this article, or claim that may be made by its manufacturer, is not guaranteed or endorsed by the publisher.
